# 3D computational models explain muscle activation patterns and energetic functions of internal structures in fish swimming

**DOI:** 10.1371/journal.pcbi.1006883

**Published:** 2019-09-05

**Authors:** Tingyu Ming, Bowen Jin, Jialei Song, Haoxiang Luo, Ruxu Du, Yang Ding

**Affiliations:** 1 Beijing Computational Science Research Center, Haidian District, Beijing, China; 2 Department of Mechanical and Automation Engineering, Chinese University of Hong Kong, Hong Kong SAR, China; 3 Department of Mechanical Engineering, Vanderbilt University, Nashville, Tennessee, United States of America; University of Cambridge, UNITED KINGDOM

## Abstract

How muscles are used is a key to understanding the internal driving of fish swimming. However, the underlying mechanisms of some features of the muscle activation patterns and their differential appearance in different species are still obscure. In this study, we explain the muscle activation patterns by using 3D computational fluid dynamics models coupled to the motion of fish with prescribed deformation and examining the torque and power required along the fish body with two primary swimming modes. We find that the torque required by the hydrodynamic forces and body inertia exhibits a wave pattern that travels faster than the curvature wave in both anguilliform and carangiform swimmers, which can explain the traveling wave speeds of the muscle activations. Notably, intermittent negative power (i.e., power delivered by the fluid to the body) on the posterior part, along with a timely transfer of torque and energy by tendons, explains the decrease in the duration of muscle activation towards the tail. The torque contribution from the body elasticity further clarifies the wave speed increase or the reverse of the wave direction of the muscle activation on the posterior part of a carangiform swimmer. For anguilliform swimmers, the absence of the aforementioned changes in the muscle activation on the posterior part is consistent with our torque prediction and the absence of long tendons from experimental observations. These results provide novel insights into the functions of muscles and tendons as an integral part of the internal driving system, especially from an energy perspective, and they highlight the differences in the internal driving systems between the two primary swimming modes.

## Author summary

For undulatory swimming, fish form posteriorly traveling waves of body bending by activating their muscles sequentially along the body. However, experimental observations have shown that the muscle activation wave does not simply match the bending wave. Researchers have previously computed the torque required for muscles along the body based on classic hydrodynamic theories and explained the higher wave speed of the muscle activation compared to the curvature wave. However, the origins of other features of the muscle activation pattern and their variation among different species are still obscure after decades of research. In this study, we use 3D computational fluid dynamics models to compute the spatiotemporal distributions of both the torque and power required for eel-like and mackerel-like swimming. By examining both the torque and power patterns and considering the energy transfer, storage, and release by tendons and body viscoelasticity, we can explain not only the features and variations in the muscle activation patterns as observed from fish experiments but also how tendons and body elasticity save energy. We provide a mechanical picture in which the body shape, body movement, muscles, tendons, and body elasticity of a mackerel (or similar) orchestrate to make swimming efficient.

## Introduction

During the undulatory swimming of fish, a backward-traveling wave of body bending is formed to push against the water and generate propulsion. Muscle is the executor of the neural control and the source of mechanical power in fish swimming. Therefore, how muscles are used is a key question in understanding the control and mechanics of fish swimming and has been a focus multidisciplinary research over the past decades.

Experimentally, muscle activation during swimming is measured using electromyography (EMG) for various fish species ([1–5], for a review see [6]). During steady swimming, a common pattern emerges: the muscle elements are activated as a wave traveling posteriorly, but this EMG wave travels faster than the curvature wave. Consequently, the phase difference between the curvature and EMG waves varies along the body, which is known as “neuromechanical phase lags”. Nonetheless, the details of the muscle activation pattern vary among species. For anguilliform swimmers such as eels, the speed difference is not large, and the duration of the muscle activation on one side of the body is approximately half of the undulation period [3]. For carangiform swimmers such as carp, the propagation speed of the EMG onset is much higher than that of the curvature wave, whereas that of EMG termination is even higher, resulting in a decrease in duration towards the tail [4]. The EMG activity, along with the muscle contraction kinetics, the strain and the volume of the active muscle, can determine the absolute muscle power output along the body. With this approach, Rome *et al*. [5] showed that for scup, the power is generated mostly by the posterior part of the body.

To understand the muscle activation patterns and underlying mechanical principles of internal driving, researchers previously studied the internal torque and the corresponding power required. The sign of the torque has been used to predict which side of the muscle should be activated. Using theoretical models, namely resistive force theory [7], elongated body theory [8], and 3D waving plate theory [9, 10], previous studies obtained torque waves that travel faster than the curvature waves and qualitatively explained the neuromechanical phase lag. However, since positive and negative torques both occupy half of the period throughout the body, the decrease in the EMG duration in carangiform swimmers remains an obscure phenomenon.

Another approach used to understand the internal driving in the coupled system is to use neural control signals as an input and observe the kinematics emerging from the coupling of internal driving, the body, and the external fluid. Using resistive force theory and 2D computational fluid dynamics (CFD) with a prescribed uniform muscle activation, McMillen *et al*. [11] and Tytell *et al*. [12] studied lamprey-like swimmers and showed that the same muscle forces can generate body bending with different wavelengths, corresponding to varying magnitudes of the neuromechanical phase lags, depending on passive body properties such as stiffness. However, since the kinematics emerge from the coupling of many components, this type of approach may generate kinematics that do not match the experimental observations; therefore, the approach may cause difficulties for systematically studying the features of muscle activation and for explaining the differences in the muscle activation between species.

These previous modeling studies were all based on either theoretical models with strong assumptions or 2D CFD models, which cannot capture 3D flow around the top and bottom of the fish body and the jet left behind and 3D shapes for carangiform swimmers [13]. Therefore, while the qualitative explanations from the models are reasonable, the errors in these predictions are difficult to estimate.

To study the features of muscle activations among different species and elucidate the underlying mechanical principles, we use 3D CFD simulations to investigate the torque patterns and power output patterns for a typical anguilliform swimmer and a typical carangiform swimmer. By combining the simulation results with experimental observations, we aim to explain the features and their variations in the EMG patterns among fish with different swimming modes.

## Model and numerical methods

**Fig 1:**
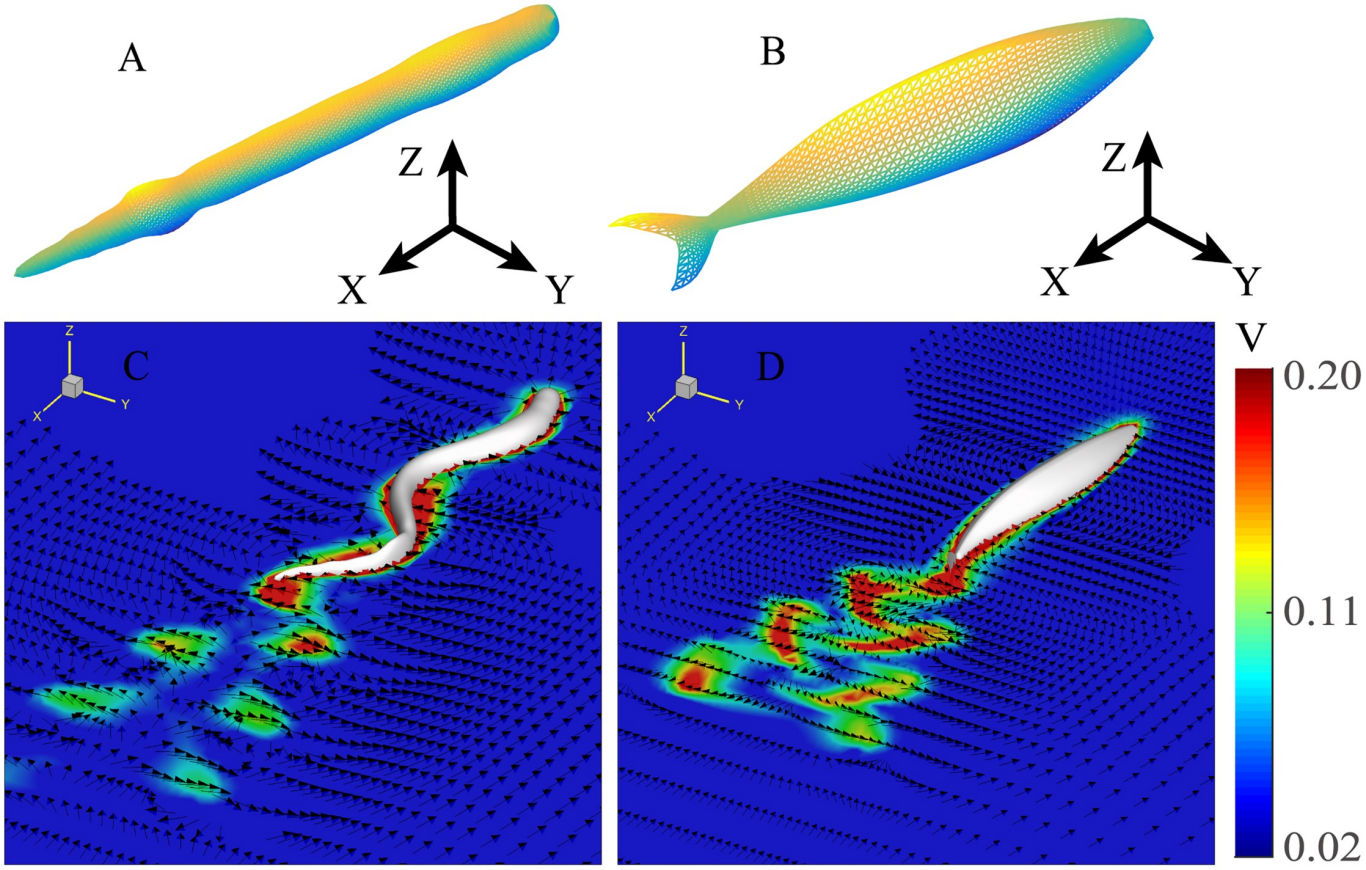
Numerical models. (A) and (B) are the meshes without body deformation for the eel and mackerel, respectively. Flow fields in the middle coronal planes (*z* = 0) of the eel (C) and mackerel (D) models in the laboratory frame. The arrows indicate the velocity direction and the colors represent the magnitude of the velocity. Only flow speeds greater than 0.02 in nondimensionalized units are shown in color.

Treating water as an incompressible viscous fluid and the fish as moving bodies with prescribed deformations, we developed two-way coupled 3D models for the swimming of an eel and a mackerel (see Fig 1).

### Body shape and kinematics

The carangiform body is modeled based on the actual anatomy of a mackerel, whereas the anguilliform body is created from a lamprey computed tomography (CT) scan (see [14] for details). Except for the caudal fin, the fins are neglected for the swimmers. The lengths of the fish bodies (*L*) are used as the unit length in the simulations. The bodies are meshed with triangular elements, and some sharp and small structures from the scan are removed to avoid instability in the CFD computation. After obtaining the surface data of the two fish, we reshaped the fish and remeshed the surface grid such that our code could accommodate the boundary between the fish and the fluid. The sharp and thin tail of the mackerel was modeled as a zero-thickness membranous structure. The number of surface mesh points was 3962 for the eel and 2127 for the mackerel (including 1962 for the mackerel’s body and 165 for the tail). The body mass (*M*) was computed by assuming a uniform distribution of density equal to the fluid density and was 1 in nondimensional units. *M* = 0.0019 for the eel and *M* = 0.0101 for the mackerel.

The kinematics for undulatory locomotion are generally in the form of a posteriorly traveling wave with the largest wave amplitude at the tail. To describe the deformation of the fish bodies, centerline curvatures *κ* are prescribed in the form of *κ*(*s*, *t*) = *A*(*s*) sin(*ks* − *ω*_*u*_*t*), where *s* is the arc length measured along the fish axis from the tip of the fish head, *A*(*s*) is the amplitude envelope of curvature as a function of *s*, *k* is the wavenumber of the body undulations that corresponds to wavelength λ, and *ω*_*u*_ is angular frequency. We use the undulation period as the unit of time; thus, *ω*_*u*_ = 2*π*. The amplitude envelope *A*(*s*) for the anguilliform kinematics has the form *A*(*s*) = *a*_max_*e*^*s*−1^, where *a*_max_ is the tail-beat amplitude. For carangiform kinematics, the amplitude envelope has the form *A*(*s*) = *a*_0_ + *a*_1_*s* + *a*_2_*s*^2^. The parameters for *A*(*s*) were adjusted to fit the envelope of the movement of real fish observed in experiments [8, 15]. The parameters used were *a*_max_ = 11.41, and *k* = 2*π*/0.59 for the anguilliform swimmer and *a*_0_ = 1, *a*_1_ = −3.2, *a*_2_ = 5.6, and *k* = 2*π*/1.0 for the carangiform swimmer. To avoid generating spurious forces and torques in the interaction between the fish bodies and fluid, we added rotation and translation in the body frame of the swimmers to ensure that the movement of the bodies without external forces satisfied two conservation laws: linear momentum conservation and angular momentum conservation (see S1 Appendix for details). The resulting kinematics are shown in S1 Fig.

### CFD and the fluid-structure interaction

The in-house immersed boundary method code that is used is capable of simulating 3D incompressible, unsteady, and viscous flows in a domain with complex embedded objects including zero-thickness membranes and general 3D bodies [16, 17]. The flow is computed on a nonuniform Cartesian grid in *x*′*y*′*z*′ coordinates. The fluid domain has a size of 8.5 × 5 × 5, and a total of 620 × 400 × 400 ≈ 99 million points are used. The grid is locally refined near the body, with the finest spacing being 0.005 × 0.005 × 0.005. The fish models are placed in the center of the computational domain, and the body centerlines are in the *z*′ = 0 plane. A homogeneous Neumann boundary condition is used for the pressure at all boundaries. The flow speeds of the inlet flow at the front boundary is set as the swimming speed in the trial runs such that the model swimmers move only minimally in the computational domain. A zero-gradient boundary condition is used at all other boundaries. At the surface of the swimmers, nonslip boundary conditions are enforced. The time interval for the integration is 5 × 10^−4^.

Because the deformations of the bodies are prescribed, there are 6 degrees of freedom for the overall movement of the swimmers, the same as that of a rigid body. We computed the 3 degrees of freedom in the 2D plane of undulation from the fluid-structure interaction, namely, forward translation, lateral translation, and yaw motion. The velocity components of the swimmers are numerically integrated at the same time interval as the CFD based on Newton-Euler equations with forces and torques from the CFD. Those 3 degrees of freedoms related to the vertical direction are neglected but the force magnitude in the direction perpendicular to the plane of motion is on average less than 1/10 of the force magnitude in the plane of motion. Because the bodies of the swimmers are deforming, the governing equation for the angular degree of freedom is d(*Iω*)/d*t* = *T*_tot_, where *I* is the moment of inertia, *ω* is the angular speed of the body, and *T*_tot_ is the total torque computed by integrating the contributions from the hydrodynamic forces on the surface of the swimmer. Since the deformation is prescribed, *I* and $\dot{I}$ are known. Therefore, *ω* can be obtained by numerically integrating $\dot{\omega }=\left(T_{\text{tot}}-\dot{I}\omega \right)/I$ while integrating other equations for the translational movement of the body and the flow of the fluid.

We set an initial swimming speed of 0.3 at the beginning of the simulation and waited at least two full cycles for the swimmer to reach steady swimming. All the data presented are collected from two periods. Because the swimming direction is not perfectly aligned with the -*x*′-axis of the computation grid, a new coordinate system is used such that the swimming direction is aligned with -*x*, *y* is the lateral direction, and the *z*-axis is the vertical direction. The Reynolds number is defined as *Re* = *UL*/*ν*_*k*_, where *U* is the swimming speed, and *ν*_*k*_ = 1/15000 is the kinematic viscosity.

### Force, torque, and power in the simulation

The force, internal torque, and power distributions along the fish body as a function of time are computed from the simulation. The force per unit length on the fish body, **F**, is calculated as follows: take an arc length Δ*s* along the body centerline, and integrate all forces from every mesh point in Δ*s*; then, divide the total force by the arc length Δ*s*.

Considering the hydrodynamic forces, we compute the internal torque required to overcome the hydrodynamic forces and body inertia. The body elasticity and the other internal resistive forces are initially ignored and will be discussed later. The torque can be found by analyzing the force balance on either side of the body from the point of interest [18] and using the concept of inertial force. When the effect of acceleration on the torque of a segment is considered as inertial force (−*m*_*b*_**a**), the effective external force can be considered **F** − *m*_*b*_**a**, where *m*_*b*_ is the body mass per unit length, $a=\dot{v}$ is the acceleration of the body segment, and the body is in static equilibrium. Then from the torque balance equation on either side of the body from the point of interest, we obtain the torque at the point of interest: $T_{\text{posterior}}\left(s,t\right)=-e_{z}\int_{s}^{1}r\times \left(F-m_{b}a\right)\text{d}l$ or $T_{\text{anterior}}\left(s,t\right)=e_{z}\int_{0}^{s}r\times \left(F-m_{b}a\right)\text{d}l$, which is consistent with [9]. Although *T* = *T*_posterior_ = *T*_anterior_ theoretically, the relative error becomes significant at the ends where torques are small. To minimize the numerical error, we use a weighted average of the torques computed from both sides, namely, *T* = *sT*_posterior_ + (1 − *s*)*T*_anterior_.

The internal power by the torque and the power transferred to the fluid per unit length are computed as $P_{T}(s,t)=T\dot{\kappa }$ [8] and *P*_*F*_ (*s*, *t*) = −**F** ⋅ **v**, respectively, where $\dot{\kappa }$ is the time derivative of curvature, and **v** is the velocity of the body segment. The difference between the total power computed by integrating the internal power and the external power along the body is within the numerical error (< 3%).

We varied the kinematics (amplitude and wavelength) and the body shape (height and width) by 10% to examine the sensitivity of the results. We found that the force and torque patterns are qualitatively the same in these tests. Simulations with a smaller mesh size result in forces and torques within the numerical error. The detailed of the test parameters and the results are provided in S2 Appendix.

## Results and discussion

### Body movement and fluid flow

The free swimming speeds (*U*) are 0.285±0.004 and 0.245±0.005 in nondimensionalized units for the eel and the mackerel, respectively. The corresponding Strouhal numbers are 0.63 and 0.65. These values are consistent with previous numerical studies at similar Reynolds numbers (*Re* ≈4000) (e.g., [14]). For both fish, double row vortices are shed behind the tail, similar to previous numerical results (see Fig 1). The velocity field behind the mackerel clearly shows a backward flow, while a mean flow behind the eel in the fore-aft direction is not easily detected.

### Force

**Fig 2:**
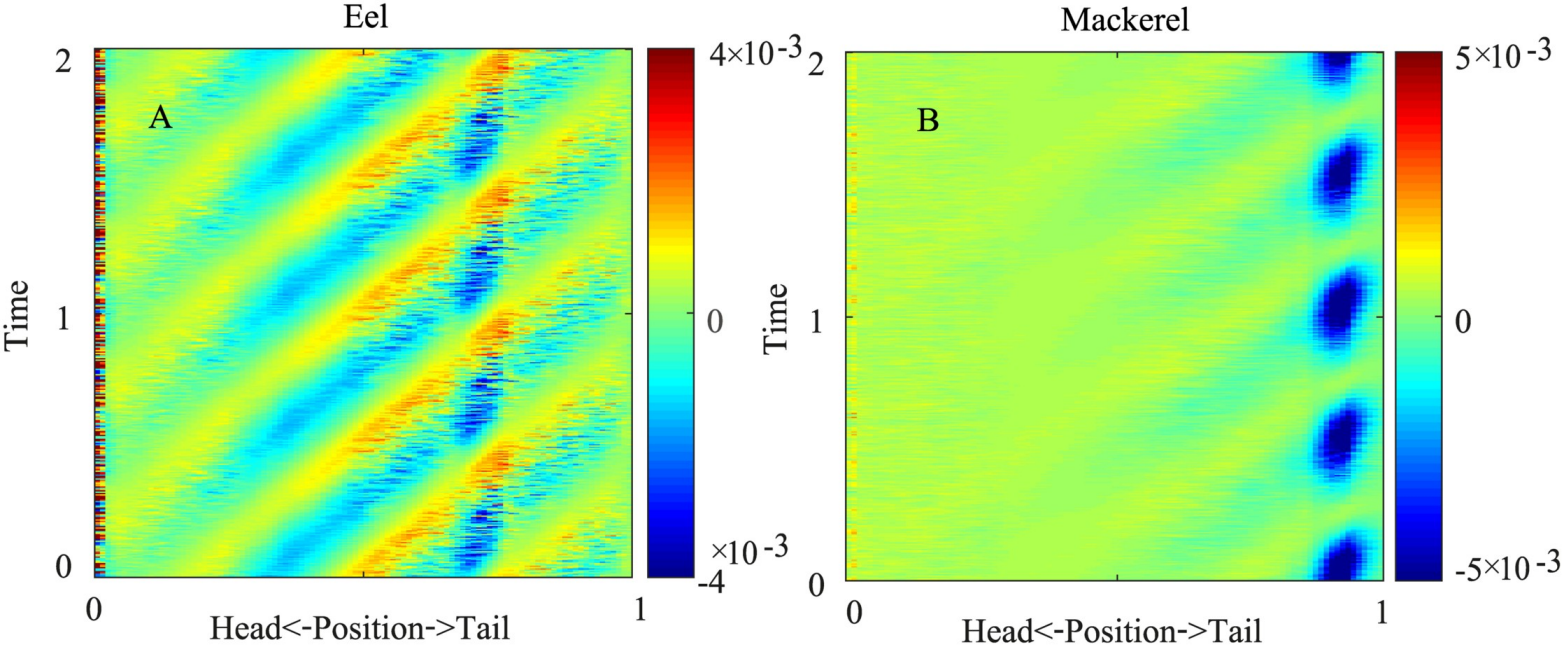
Spatiotemporal distribution of the fore-aft force (*F*_*x*_) on the eel (A) and the mackerel (B) for two periods. Negative values indicate thrust, because the swimming direction is in the -*x* direction.

**Fig 3:**
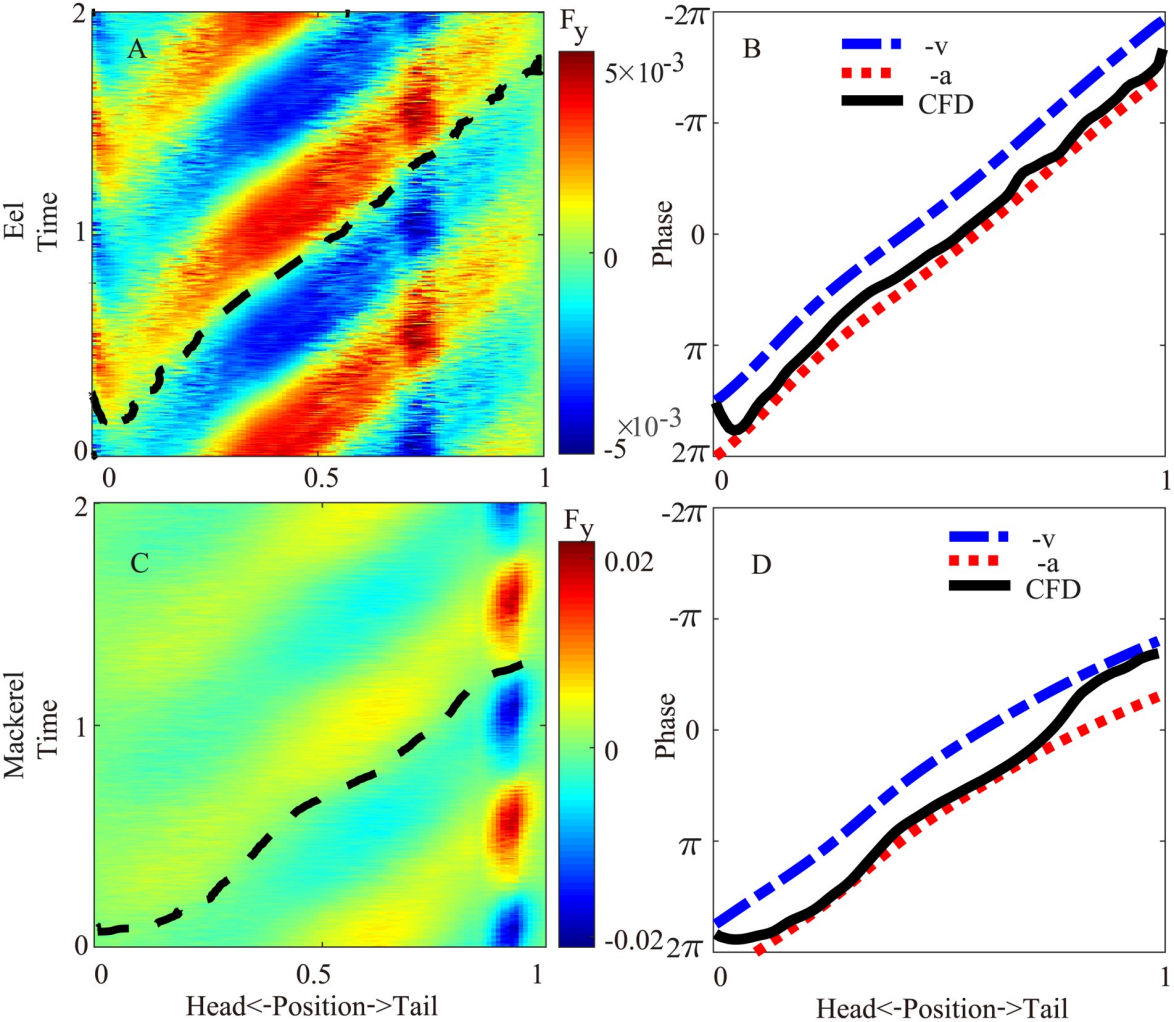
Lateral force (*F*_*y*_). (A & C,) Spatiotemporal distribution of the lateral force on the body for two periods from the simulation. The dashed black line indicates a zero-crossing (phase) of the force. (B & D) Comparison of the phase of the lateral force along the body from CFD (solid black line, the same as the dashed lines in A and C) with the phase of the negation of the velocity and the phase of the negation of the acceleration. A 2*π* term is added or subtracted to ensure continuity.

As expected from the input kinematics and body shapes, the forces are relatively uniformly distributed on the eel but concentrated on the tail of the mackerel (Figs 2 and 3A & 3C, S1 and S2 Videos). The fore-aft and lateral forces both show posteriorly traveling wave patterns similar to those of body bending, except at the head where the surface orientation rapidly changes. For the eel, the peaks in the force components near 0.7 body length correspond to an increase in the body height (in *z* direction) at that position. For the mackerel, the separation of the thrust and drag is clear: the tail generates most of the thrust, and the anterior part of the body generates drag at all times.

Because the phase of the force, especially the lateral force, is essential in determining the phase of the torque [18], we compare the phases of the lateral forces from the simulation with those of the velocity and the acceleration of the segments. In general, if the lateral force from the fluid on a segment is in phase with the negation of the segment velocity, it is a resistive-like force, and if the lateral force is in phase with the negation of the acceleration of the segment, it is a reactive-like force. We find that the phase of the observed lateral force on the body is closer to the phase of the negation of the acceleration except near the snout tips and the tail for the mackerel. In these regions, the phase of the lateral force is close to the negation of the velocity. In general, the forces on the fish are close to the predicted forces from elongated body theory, but discrepancies exist when the shape changes are rapid. Detailed discussions of the hydrodynamics underlying the force pattern are beyond the scope of this paper.

### Torque

**Fig 4:**
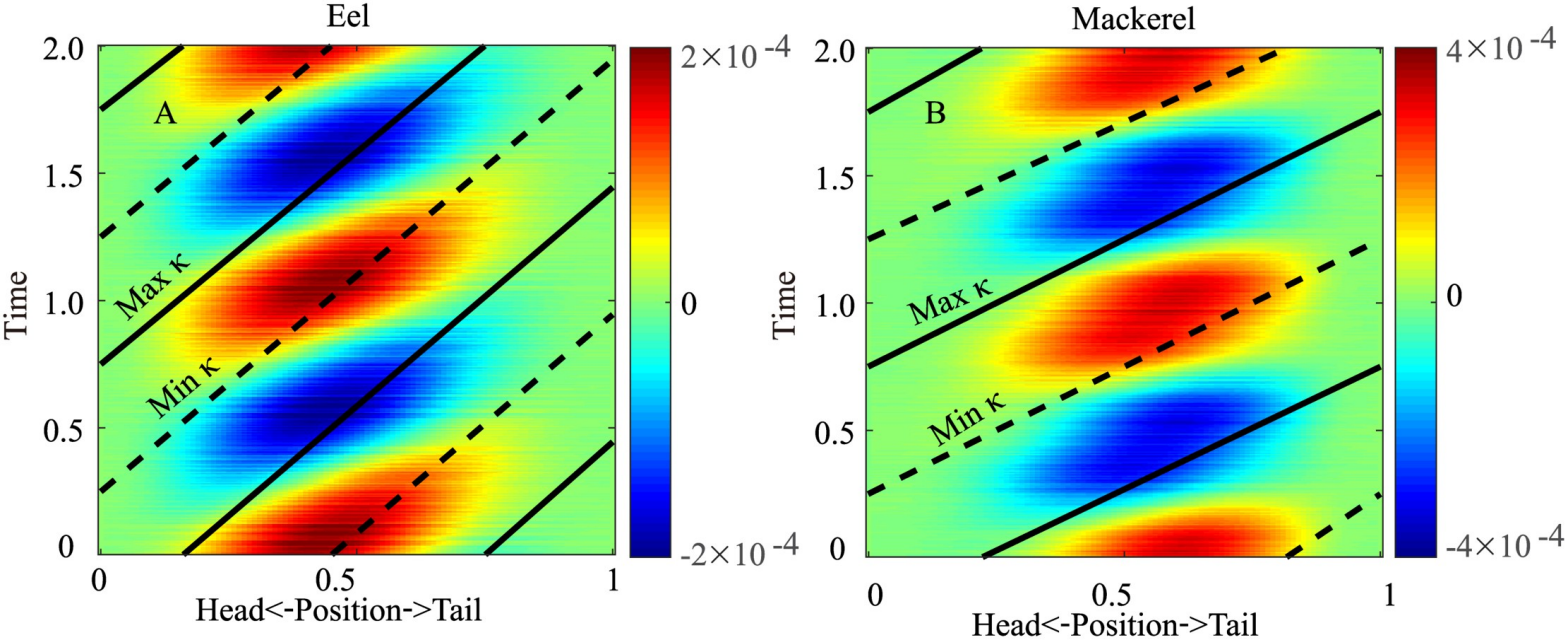
Spatiotemporal distribution of the torque on the body in two periods for the eel (A) and the mackerel (B). The solid and dashed lines indicate the maximum and minimum curvatures, respectively. The same information is illustrated in S3 and S4 Videos.

The torque required to overcome the hydrodynamic forces and body inertia in both species exhibits a traveling wave pattern moving posteriorly with a higher speed than the curvature wave (Fig 4). For the eel, the average speed of the torque wave (*v*_*T*_) is 1.41 in the nondimensionalized unit (body length/period). The traveling wave speed of the torque is even higher in the mackerel (*v*_*T*_ = 2.11), exhibiting a nearly standing wave pattern. The torque wave speeds qualitatively match the observation that the EMG speed is much higher in carangiform swimmers [6]. The maximal value of the torque appears at approximately the middle of the body of the eel and slightly posterior to the middle point for the mackerel.

### Power

**Fig 5:**
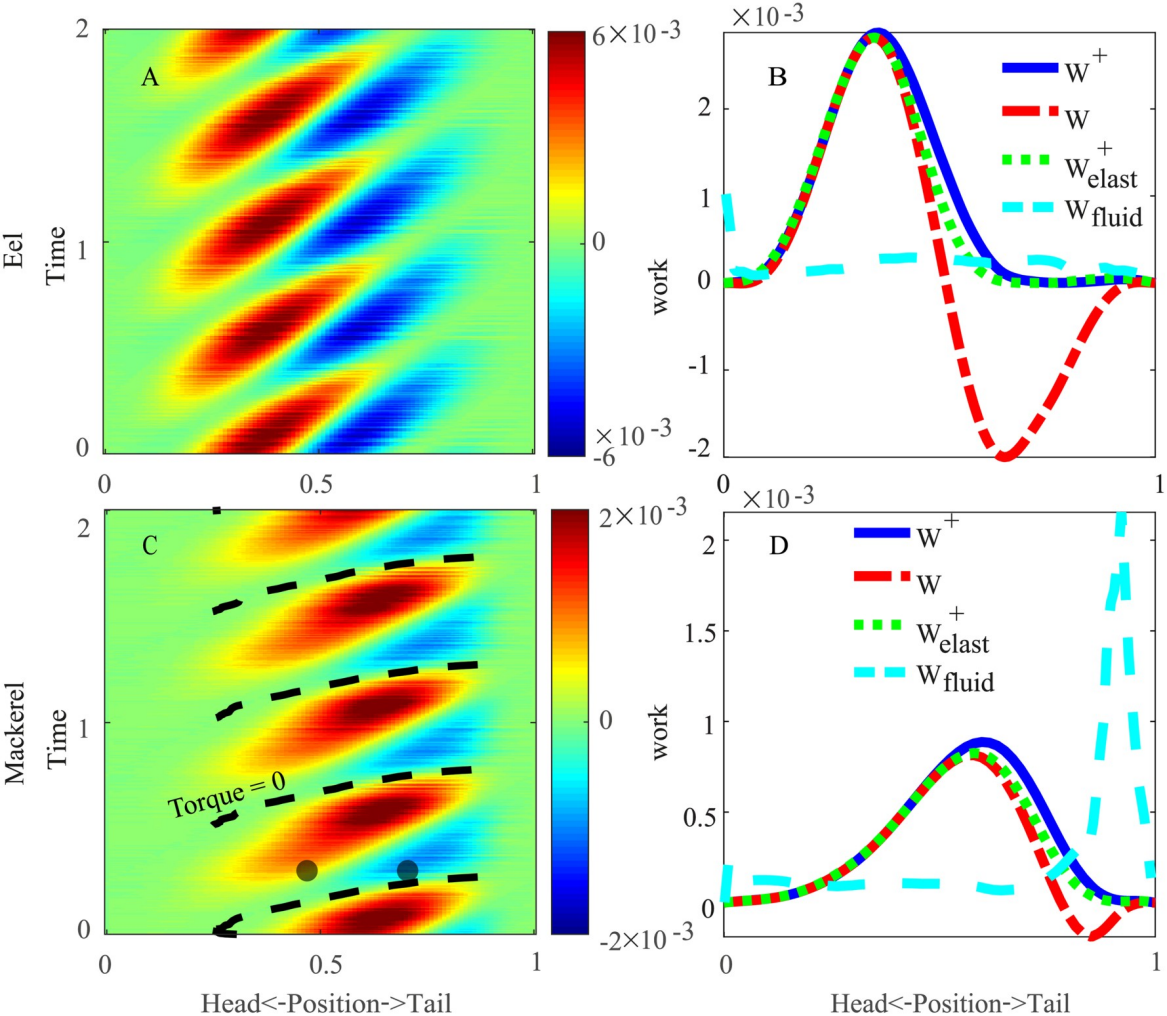
Internal power (*P*_*T*_(*s*, *t*)) distribution for the eel (A) and the mackerel (C) and the work done over a cycle by muscles along the bodies of the eel (B) and the mackerel (D). The dashed line in (C) indicates the zero-crossing of the torque in the mackerel (Fig 4B). The two black dots in (C) indicate an example time instant when two points on the body have the opposite sign of power but the same sign of torque. The solid blue lines in (B & D) represent the work by integrating only the positive values in (A & C), and the dashed-dotted red lines represent the work (*W*) by integrating both positive and negative values. The dotted green lines represent the positive work *W*^+^ when body elasticity is considered (Fig 7B & 7D). The cyan dashed lines represent the work done to the fluid by the integration of *P*_*F*_(*s*, *t*).

As shown in Fig 5, the power from the torque is mostly positive, indicating the energy output from the muscle, but negative values are observed on the posterior parts of both fish. For the eel, the power is nearly all negative for *s* > 0.7, similar to the case with a floppy body in a previous 2D study [12], whereas for the mackerel, the negative power is intermittent on the posterior part. The work over a cycle calculated by simply integrating the power is the minimal work needed, because the dissipation due to the internal resistance is not included; this method implies that the negative power transferred to the body is fully stored and recovered. The peak of this work per cycle is at the anterior part (≈0.4) for the eel and at a more posterior position for the mackerel (≈0.58), slightly posterior to the peak magnitude of the torque. We find that the work over a cycle is significantly negative on the posterior half of the eel body and slightly negative near the tail of the mackerel. If we assume that no energy-storing and energy-transmitting elements exist, then the work done by the muscles is the integration of only the positive power. We denote this quantity by *W*^+^. The differences between the two types of work per cycle are the greatest for the posterior part of the eel, indicating that power is lost if no spatial energy transfer is performed inside the body of the eel. The distribution of power transferred to the fluid from the body is relatively uniform on the eel but concentrated on the tail of the mackerel (cyan dashed lines in Fig 5B & 5D).

The mean total power *P*_tot_ averaged over a cycle is 2.2 × 10^−4^ (in nondimensionalized units) for the eel and 2.6 × 10^−4^ for the mackerel. If only the positive power is used, the power becomes $P_{\text{tot}}^{+}=8.6\times 10^{-4}$ and $P_{\text{tot}}^{+}=3.4\times 10^{-4}$, for the eel and the mackerel, respectively. The significant differences between *P*_tot_ and $P_{\text{tot}}^{+}$ indicate a great potential to improve energetic efficiency through the spatiotemporal transfer of energy.

### Understanding the torque and power patterns

**Fig 6:**
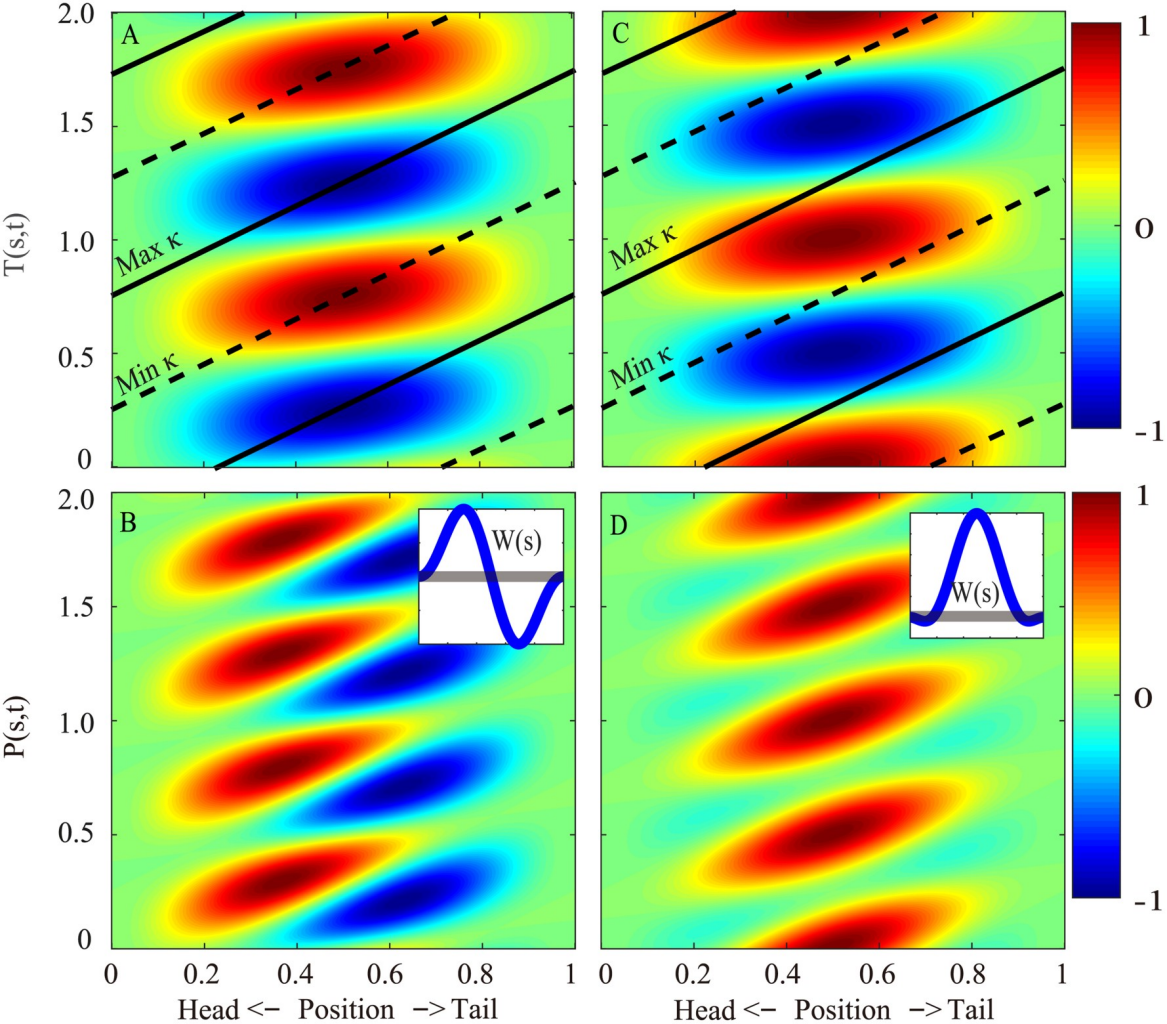
Spatiotemporal distributions of the torque (*T*) and power (*P*) along the body when pure resistive forces (A for *T* and B for *P*) or pure reactive forces (C for *T* and D for *P*) are considered. All values are normalized to the respective maximum values in each subfigure. The solid and dashed lines indicate the maximum and minimum curvatures, respectively. The insets are the distribution of the work ($W\left(s\right)=\int_{0}^{1}P\left(s,t\right)\text{d}t$) done by the internal torque. The gray lines in the insets indicate 0 to guide the eye. In the calculation, the body is uniform, the undulation amplitude is uniform and infinitesimal, and the wavelength is the same as the body length. See S3 Appendix for details of the derivation.

The torque pattern can be understood by applying the results obtained in a previous study [18]: the torque pattern in undulatory locomotion is determined mainly by the wavelength and phase of the lateral force relative to the lateral movement. The torque wave of the eel has a relatively low wave speed compared to that of the mackerel due to the short wavelength of the undulation. Because the phase of the force for the eel is overall close to the phase of the reactive force, the internal torque and power patterns are also similar to the patterns associated with pure reactive forces (Fig 6, left column). For the mackerel, the long wavelength of the curvature wave and the concentrated force on the tail result in nearly synchronized torques on the body. Because the force from the tail to the fluid is nearly in phase with the velocity, the rate of change of the curvature ($\dot{\kappa }$) and the torque are also nearly in phase. Consequently, the torque and power patterns are similar to the patterns associated with pure resistive forces (Fig 6, right column), and the internal power is nearly all positive.

### Body viscoelasticity explains the wave speed variation of EMG

**Fig 7:**
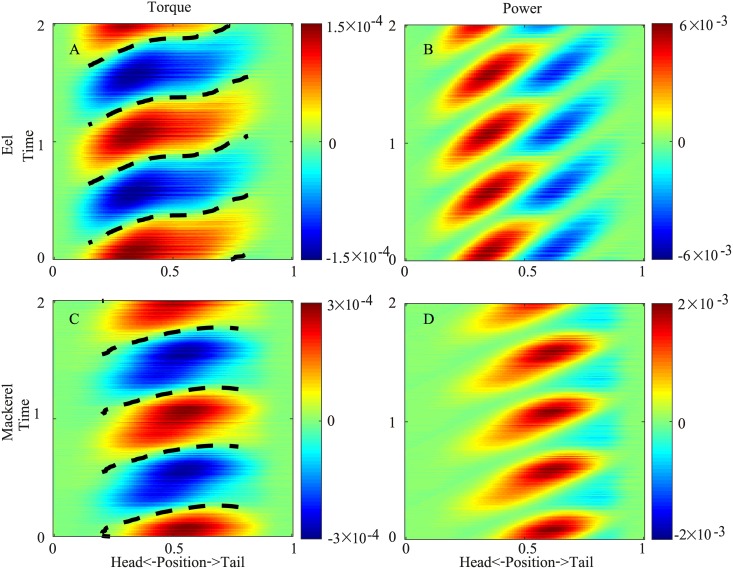
Torque (A) and power (B) distributions when the elasticity of the body is considered for the eel and torque (C) and power (D) distributions for the mackerel. The dashed lines indicate zero-crossings of the torque.

Previous bending tests and experiences in the handling of fish indicate that the torque from the viscoelasticity of an eel body is significant but smaller than the torque generated by muscles [19]. For carangiform swimmers, since no muscles exist behind the peduncle region and the curvature is comparable (albeit greater) to the rest of the body, the torque from elasticity must be significant at least in the tail region. However, an accurate *in vivo* measurement of the body viscoelasticity distribution is not available. Therefore, we discuss the trend of the influences of the viscosity and elasticity individually when the elasticity or viscosity is small relative to the torque from hydrodynamics and the body inertia (Fig 7).

We assume that the magnitude of the torque from the body elasticity or viscosity is 40% of the torque at individual positions along the body, namely *T*_*e*_ = 0.4〈*T*〉*κ*(*s*, *t*)/〈*κ*〉 or $T_{v}=0.4\langle T\rangle \dot{\kappa }\left(s,t\right)/\langle \dot{\kappa }\rangle$, where “〈〉” means standard deviation over time. Then, the total torque that needs to be generated becomes *T* + *T*_*e*_ or *T* + *T*_*v*_. As shown in Fig 7, we find that the effect of elasticity on the torque is different along the body, separated by a position (*s* ≈ 0.5 for the mackerel and *s* ≈ 0.3 for the eel) where *T* and $\dot{\kappa }$ are in phase and the power is all positive. Anterior to that point, the torque magnitudes increase, and the torque wave speeds decrease; posterior to that point, the torque magnitude decreases, and the speed of the torque wave increases. For the mackerel, the torque wave can even reverse when the phase shift effect of the elasticity is strong. The reversal of the wave resembles the reversal of the wave of the offset of the EMG observed for carangiform swimmers [6]. As a result of the changes in the torque, the area of the negative power region in the posterior part of the body decreases, and *W*^+^ decreases (Fig 5D). This observation is consistent with the findings of previous studies that suitable elasticity can save and restore energy to improve efficiency (e.g., [20]). For the eel, the effect of the speed increase ends near *s* = 0.7 when the maximal curvature coincides with the minimal torque without elasticity. Therefore, the energy storage and release for the eel is in the middle part of the body (Fig 5B). Since the body viscoelasticity of the eel is weak and this effect is subtle, changes in the wave speed of the middle part of the torque wave or EMG are not obvious.

**Fig 8:**
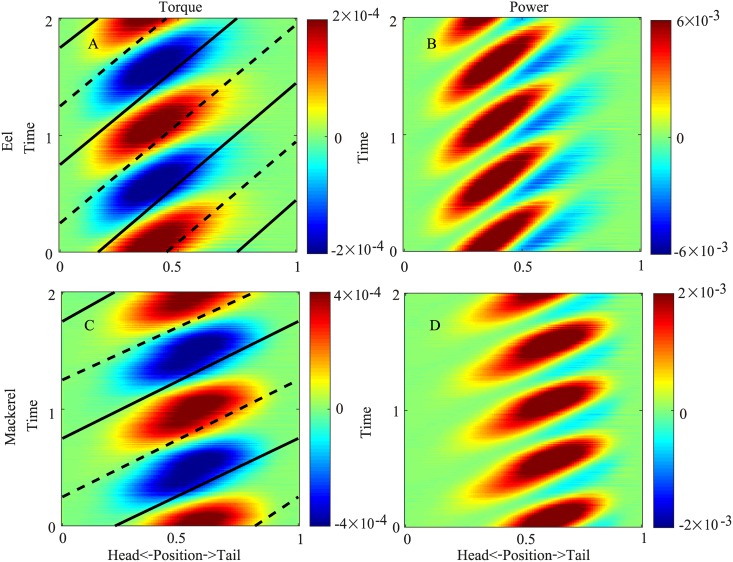
Torque (A) and power (B) distributions when the viscosity of the body is considered for the eel and torque (C) and power (D) distributions for the mackerel. The solid and dashed lines indicate the maximum and minimum curvatures, respectively.

Since the body viscosity requires a torque that is in phase with the time derivative of the curvature, for both the eel and the mackerel, the resulting torques become more aligned with the time derivative of the curvature and hence have wave speeds closer to the speed of the curvature (Fig 8). Consequently, the negative power regions are reduced because the viscosity of the body always dissipates energy. The effects of the viscosity and elasticity are qualitatively the same as greater contributions, at least to a prefactor of 0.8 for *T*_*e*_ and *T*_*v*_ (see S1 Appendix for details).

### Tendon connection explains the duration decrease of EMG

Although local elasticity can temporally transfer the energy flow into this region due to the fluid-structure interactions, the spatial transmission of such energy can only be achieved by other structures. In animals, coupled joint articulation by tendons over two or more joints is common and is an effective structure to save and transfer energy by connecting a joint with positive power and another joint with negative power [21, 22]. For carangiform swimmers, long tendons exist that span many vertebra [23]. Although the force transfer function of these tendons towards the tail has been experimentally confirmed, to our best knowledge, their function in saving energy has not been considered. We hypothesize that these long tendons are used to transfer energy from the posterior part to the middle part of the body when the negative power appears on the posterior part (Fig 5). This hypothesis can explain the observed decrease in the muscle activation duration among the carangiform swimmers, including some detailed features: the increase in the duration of the negative power from the middle of the body towards the tail matches the decrease in the EMG duration. The start of the positive power is aligned with the sign change of $\dot{\kappa }$ (the lines in Fig 4B), resulting in a low speed that is the same as that of the curvature wave. The end of the positive power is aligned with the sign change in the torque (the dashed lines in Fig 5B), resulting in a high speed that is the same as that of the torque wave. Such differences in wave speed qualitatively match the speed differences of the onset and offset of the EMG. Note that this hypothesis does not contradict the common view that force and energy are transmitted to the tail to interact with the fluid. Torque is still required when the power is negative on the posterior region and can be provided by the muscle in a more anterior position connected by the tendon. This hypothesis is also consistent with the observation that the EMG duration is nearly half of the undulation period on the whole body of anguilliform swimmers, which do not possess long tendons [23].

**Fig 9:**
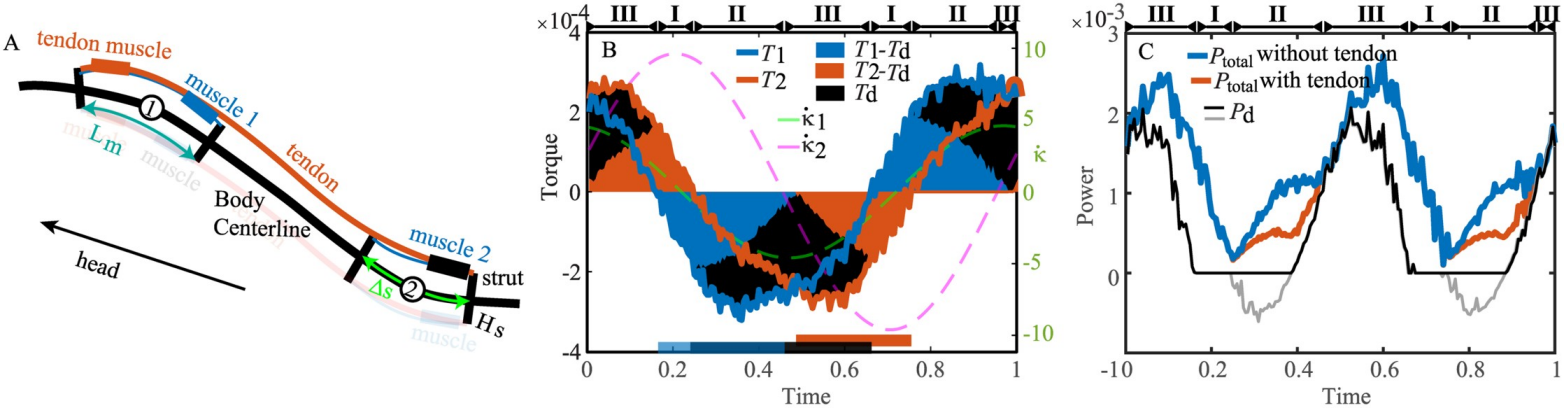
The energy transfer by tendons. (A) Diagram of the tendon model. The muscles and tendon on the left side are drawn as semitransparent for clarity. (B) The torques and time derivative of curvatures as a function of time at the two joints and the torque generated by the tendon muscle. The vertical lengths of the shaded regions indicate the magnitudes of the respective torques. The bars at the bottom indicate the predicted muscle activation periods when the tendons are used. The blue and black bars combined predict the muscle activation at joint 1, and the red bar predicts the muscle activation at joint 2. (C) The total power generated by the muscles with and without the tendons and the power from the tendon muscle. The gray lines indicate the negative power on the tendon muscle but is counted as 0 in computing *P*_total_. The stage indices are labeled at the tops of (B) and (C).

The energy transfer and savings by a tendon and the shortening of muscle activation can be further elucidated by a simplistic rope model (Fig 9). We take the positions *s*_1_ = 0.47 and *s*_2_ = 0.71 on the mackerel as an example. We assume that the designated muscles (muscle 1 and muscle 2 in Fig 9A) are attached to virtual struts with a height *H*_*s*_. Based on experimental observations on the arrangement of muscles, tendons and vertebral segments in the posterior part of carangiform swimmers [24, 25], a pair of muscles on anterior position are connected to the posterior point by tendons (hereafter referred to as ‘tendon muscles’). A simple relationship between the muscle force *F*_*m*_ and the torque about the points on the centerline from a muscle *T*_*m*_ can be derived: *T*_*m*_ = *H*_*s*_*F*_*m*_. Correspondingly, the change in the muscle length and the curvature has the following relation: Δ*L*_*m*_ = *L*_*m*_ − *L*_*m*0_ = *H*_*s*_*κ*Δ*s*, where *L*_*m*0_ is the muscle length at rest, and Δ*s* is the arc length between the struts without bending. The power per unit length can be computed as $P=F_{m}{\dot{L}}_{m}/\Delta s=T\dot{\kappa }$. This relationship also holds for the tendon muscles. Because the height of the struts, the arc distance between the struts, and the resting length of the muscle do not affect the power, the exact values of these quantities are not important in this analysis.

We consider the case where the phases of *T*_1_ = *T*(*s*_1_), *T*_2_ = *T*(*s*_2_), ${\dot{\kappa }}_{1}=\dot{\kappa }\left(s_{1}\right)$, and ${\dot{\kappa }}_{2}=\dot{\kappa }\left(s_{2}\right)$ have the relation $\phi_{\dot{\kappa }1}\approx \phi_{T1}>\phi_{T2}>\phi_{\dot{\kappa }2}$ (Fig 9B). We assume a strategy in which the tendon muscles are only active when the torque required at these two points have the same sign and one of the tendon muscles generates a torque *T*_*d*_ that is needed by both joints, namely, *T*_*d*_ = sgn(*T*_1_) min(|*T*_1_|, |*T*_2_|). We first assume that any negative power of the muscles is wasted. Then, there are four stages (see Fig 9B & 9C):

I *T*_1_ and *T*_2_ have different signs, the tendon muscle is not activated, and the total muscle power is unchanged.
II *T*_1_, *T*_2_, and ${\dot{\kappa }}_{1}$ have the same sign but opposite to ${\dot{\kappa }}_{2}$. The power from muscle 1 is $\left(T_{1}-T_{d}\right){\dot{\kappa }}_{1}$, that from muscle 2 is $\max(\left(T_{2}-T_{d}\right){\dot{\kappa }}_{2},0)=0$, and that from the tendon muscle on both joints is $P_{d}=\max\left(T_{d}\left({\dot{\kappa }}_{1}+{\dot{\kappa }}_{2}\right),0\right)=T_{d}{\dot{\kappa }}_{1}-\min(|T_{d}{\dot{\kappa }}_{1}\left|,\right|T_{d}{\dot{\kappa }}_{2}\left|\right)$. The total power is $T_{1}{\dot{\kappa }}_{1}$ without the tendon and $T_{1}{\dot{\kappa }}_{1}-\min(|T_{d}{\dot{\kappa }}_{1}\left|,\right|T_{d}{\dot{\kappa }}_{2}\left|\right)$ with the tendon. The energy saved is $\min(|T_{d}{\dot{\kappa }}_{1}|,|T_{d}{\dot{\kappa }}_{2}|)$.
III *T*_1_, *T*_2_, ${\dot{\kappa }}_{1}$, and ${\dot{\kappa }}_{2}$ all have the same sign. The power from muscle 1 is $\left(T_{1}-T_{d}\right){\dot{\kappa }}_{1}$, that from muscle 2 is $\left(T_{1}-T_{d}\right){\dot{\kappa }}_{2}$, and that from the tendon muscle is $P_{d}=T_{d}\left({\dot{\kappa }}_{1}+{\dot{\kappa }}_{2}\right)$. The tendon has no effect on the total power.
IV *T*_1_, *T*_2_ and ${\dot{\kappa }}_{2}$ have the same sign but opposite to ${\dot{\kappa }}_{1}$. Similar to case 2, the power from muscle 1 is $\max(\left(T_{1}-T_{d}\right){\dot{\kappa }}_{1},0)=0$, that from muscle 2 is $\left(T_{2}-T_{d}\right){\dot{\kappa }}_{2}$, and that from the tendon muscle is $P_{d}=\max\left(T_{d}\left({\dot{\kappa }}_{1}+{\dot{\kappa }}_{2}\right),0\right)=T_{d}{\dot{\kappa }}_{2}-\min(|T_{d}{\dot{\kappa }}_{1}\left|,\right|T_{d}{\dot{\kappa }}_{2}\left|\right)$. The power saved is $\min(|T_{d}{\dot{\kappa }}_{1}|,|T_{d}{\dot{\kappa }}_{2}|)$.

In the example, because $\phi_{\dot{\kappa }1}<\phi_{T1}$, only stages I–III are present. The energy saved is 20% of the energy output from muscle 2 without the tendon and accounts for 60% of the total negative power that can potentially be saved. The remaining 40% of the energy is wasted in the tendon muscle due to the lengthening of the tendon-muscle system at the beginning of stage II (gray curves in Fig 9C). If the tendon is elastic, then the energy could be saved during the lengthening and be released during the shortening in the later stage II to further improve efficiency. The muscle activation at point 1 comes from the combination of muscle 1 and tendon muscle, which take a half period on one side of the body, and the muscle activation at point 2 only comes from muscle 2, which takes less than a half period (see the bars at the bottom of Fig 9B).

### Error associated with *Re*

The low swimming speeds that we observed (compared with those of real animals) are likely due to the low *Re* used in our simulations. However, we argue that the results are qualitatively representative of real adult fish. First, a meta-analysis of previously reported fish swimming data indicates that the transition from the viscous regime to the turbulent regime occurs at a *Re* of several thousand [26]. Second, even the eel model in our study shows an inertia-dominated mode of swimming. Because the drag coefficient decreases with increasing *Re* in general, the speed of the simulated swimmer is expected to increase with increasing *Re*, and the contribution of the resistive force is expected to decrease for a real adult eel.

### Conclusion

Using 3D numerical models, we provide the most accurate prediction of the torque and power required for hydrodynamic forces during the undulatory swimming of fish. By considering the torque and power transfer by tendons and the body viscoelasticity, we for the first time provide explanations for some long-standing questions in muscle activation patterns: the shortening of muscle activations in carangiform swimmers and reversal of the wave of the offset of EMG. Our study offers an integrative view of the function of the muscles as part of the mechanical system, highlights the differences in the internal driving of two primary swimming modes, and provides insights into the energy transfer and energy saving mechanisms by body elasticity and tendons in undulatory swimming. The numerical models developed and the mechanisms revealed in this study may guide the design of efficient bioinspired robots, especially soft robots with distributed driving systems and elastic bodies [27, 28].

## Supporting information

S1 Appendix Description of the method to satisfy momentum conservation and obtain kinematics in the body frames. (PDF)

S2 Appendix Sensitivity and convergence tests. (PDF)

S3 Appendix Derivation of the torque and power from pure resistive forces and reactive forces. (PDF)

S1 Fig The kinematics of the swimmers in their own body frames (free movement in vacuum). (A) Eel. (B) Mackerel.
(TIF)

S1 Video The force distribution along the body of the eel. The black line represents the midline of the fish and the red arrows represent the hydrodynamic forces. The head is on the left and the tail is on the right.
(AVI)

S2 Video The force distribution along the body of the mackerel. The black line represents the midline of the fish and the red arrows represent the hydrodynamic forces. The head is on the left and the tail is on the right.
(AVI)

S3 Video The torque distribution along the body of the eel. The magnitude of the torque in the *z* direction is represented by the color.
(AVI)

S4 Video The torque distribution along the body of the mackerel. The magnitude of the torque in the *z* direction is represented by the color.
(AVI)

S5 Video The torques from simulations with different mesh sizes for the eel. (AVI)
