# Supplementary material for: 3D computational models explain muscle activation patterns and energetic functions of internal structures in fish swimming
Source: PLoS Comput Biol. 2019 Sep 5;15(9):e1006883. doi: 10.1371/journal.pcbi.1006883 (PMC6748450; doi:10.1371/journal.pcbi.1006883)
Supplement: S1 Appendix — (PDF) [file pcbi.1006883.s001.pdf]

Supplementary Information for “3D computational models explain muscle activation patterns and energetic functions of internal structures in fish swimming”

Kinematics in the body frames

Since only curvature is prescribed in our model, the position of a body segment relative to the head $\mathbf{r}_h = (x_h, y_h)$ can be computed by integrating the curvature twice:

$$\begin{aligned} \theta(s,t) &= \int_0^s \kappa(l,t)\mathrm{d}l, \\ x_h(s,t) &= \int_0^s \cos(\theta(l,t))\mathrm{d}l, \\ y_h(s,t) &= \int_0^s \sin(\theta(l,t))\mathrm{d}l. \end{aligned} \tag{S1}$$

However, linear momentum and angular momentum are not conserved if such positions are used as the segment position in its body frame without external forces. In order to ensure the conservation of the linear momentum of the fish, the center of mass (COM) is computed and the origin of the coordinates is moved to the COM, i.e. $(x', y') = (x_h - x_{\mathrm{COM}}, y_h - y_{\mathrm{COM}})$. Next, we assume that there is an angular velocity $\omega = \omega_b(t)\mathbf{e}_z$ about the COM and compute the variation of angular momentum. The angular momentum of the whole body about the COM can be computed as

$$\mathbf{L_t}(t) = L_t(t)\mathbf{e}_z = \int_0^1 m[\mathbf{r} \times (\omega_{\mathbf{b}} \times \mathbf{r} + \mathbf{v})]\mathrm{d}l, \tag{S2}$$

where $m(s)$ is the mass per unit length of the fish, $\mathbf{r}$ is the displacement of a point on the midline of the body from the COM, and $\mathbf{v} = \dot{\mathbf{r}}$. Angular momentum conservation requires $\mathrm{d}L_t/\mathrm{d}t = 0$. That is

$$0 = \int_0^1 2m\omega_b \mathbf{r}\cdot\mathbf{v} + m\dot{\omega}_b r^2 + m\mathbf{r}\times\mathbf{a}\mathrm{d}l, \tag{S3}$$

where $\mathbf{a} = \dot{\mathbf{v}}$.

ω_b is solved by a spectral method. First, ω_b is approximated by N-term truncated Fourier series: $\omega_b = \sum_{n=1}^{N}(a_n\cos(2\pi nt) + b_n\sin(2\pi nt))$. Then a_n and b_n are found by minimizing $|\mathrm{d}L_t/\mathrm{d}t|$ to the order of 10^{-10}.

The angle of rotation of the body is obtained by $\theta_b = \int \omega_b$. Every point on the body at a time instant t is rotated by $\theta_b(t)$ so that the angular momentum is conserved. An additional constant rotation is applied to align the swimming direction to the $-x$ direction of the Cartesian grid in the simulation. The resultant motions of the midlines are shown in Fig. S2.
