# Supplementary material for: 3D computational models explain muscle activation patterns and energetic functions of internal structures in fish swimming
Source: PLoS Comput Biol. 2019 Sep 5;15(9):e1006883. doi: 10.1371/journal.pcbi.1006883 (PMC6748450; doi:10.1371/journal.pcbi.1006883)
Supplement: S2 Appendix — (PDF) [file pcbi.1006883.s002.pdf]

Supplementary Information for "3D computational models explain muscle activation patterns and energetic functions of internal structures in fish swimming"

The influence of variations in geometric and kinematic parameters

To study the sensitivity of our results to the geometric and kinematic parameters, we choose four different cases for each kind of swimmer (Tab 1, Tab 2, and Fig 1). Our results show that the swimming characteristics do not sensitively depend on the geometric and kinematic parameters. We observed relatively large error in power because power scale with the third power of length. The pattern of the torque and power remain the same with varying parameters except wavelength. As discussed in the main text, the torque pattern is mainly determined by the phase of the force and the wavelength. As shown in Fig 1, the longer the wavelength, the faster the torque wave.

Table 1: The results of sensitivity tests of eel.

Case	Parameter variations		Results			
			U	P_t	P_f	St
control			0.29	2.2E-04	2.1E-04	0.63
	l_a	l_b				
geo-1	+10%	+10%	0.28	2.4E-04	2.3E-04	0.63
geo-2	-10%	-10%	0.29	2.0E-04	2.0E-04	0.62
	$a_{\max}$	λ				
kin-1	+10%	+10%	0.34	3.1E-04	3.1E-04	0.72
kin-2	-10%	-10%	0.23	1.4E-04	1.4E-04	0.63

The control case uses parameters reported in the main text. All data are collected from the third and forth cycles. l_a and l_b are the major and minor axis of cross section of the body, a_{max} is the amplitude of the centerline curvature, and λ is the wavelength. The centerline curvature is prescribed as $\kappa(s,t) = a_{\max}\exp(s-1)\sin(ks-\omega t)$.

Table 2: The results of sensitivity tests of mackerel.

	Parameter variations				Results			
case					U	P_t	P_f	St
control					0.25	2.6E-04	2.6E-04	0.64
	l_a	l_b	l_h					
geo-1	+10%	+10%	+10%		0.25	3.0E-04	3.0E-04	0.65
geo-2	-10%	-10%	-10%		0.25	2.2E-04	2.2E-04	0.64
	a_0	a_1	a_2	λ				
kin-1	+10%	0%	0%	+10%	0.27	4.1E-04	4.1E-04	0.68
kin-2	-10%	0%	0%	-10%	0.23	1.5E-04	1.5E-04	0.59

The control case uses parameters reported in the main text. All data are collected from the third and forth cycles. l_a and l_b are the major and minor axis of cross section, l_h is the height of the tail, a_0 is the amplitude of centerline curvature, and λ is the wavelength. The centerline curvature is prescribed as $\kappa(s,t) = (a_0 + a_1 s + a_2 s^2)\sin(ks-\omega t)$.

The influence of the magnitude of body viscoelasticity

To study the effect of elasticity and viscosity on the torque and power patterns, we set the pre-factors for the magnitude of torque from the body elasticity or viscosity as 0.1,0.2,0.4 and 0.8 (Fig 3 for eel & Fig 4 for mackerel). The trend is the same except when the torque from elasticity is comparable to those from hydrodynamic forces for the eel. When the prefactor is 0.8, the torque transits to a two wave like pattern. The negative power region in the posterior part of body becomes smaller when the pre-factors increases (see in Fig 3 & Fig 4), and W^+ becomes smaller too (see Fig 5).

The wave speed decreases as the pre-factor increases when viscosity is considered. Similarly, the area of negative power region becomes smaller, but the positive power region becomes greater and the amplitude increases As a result, the total energy consumption increases with viscosity.

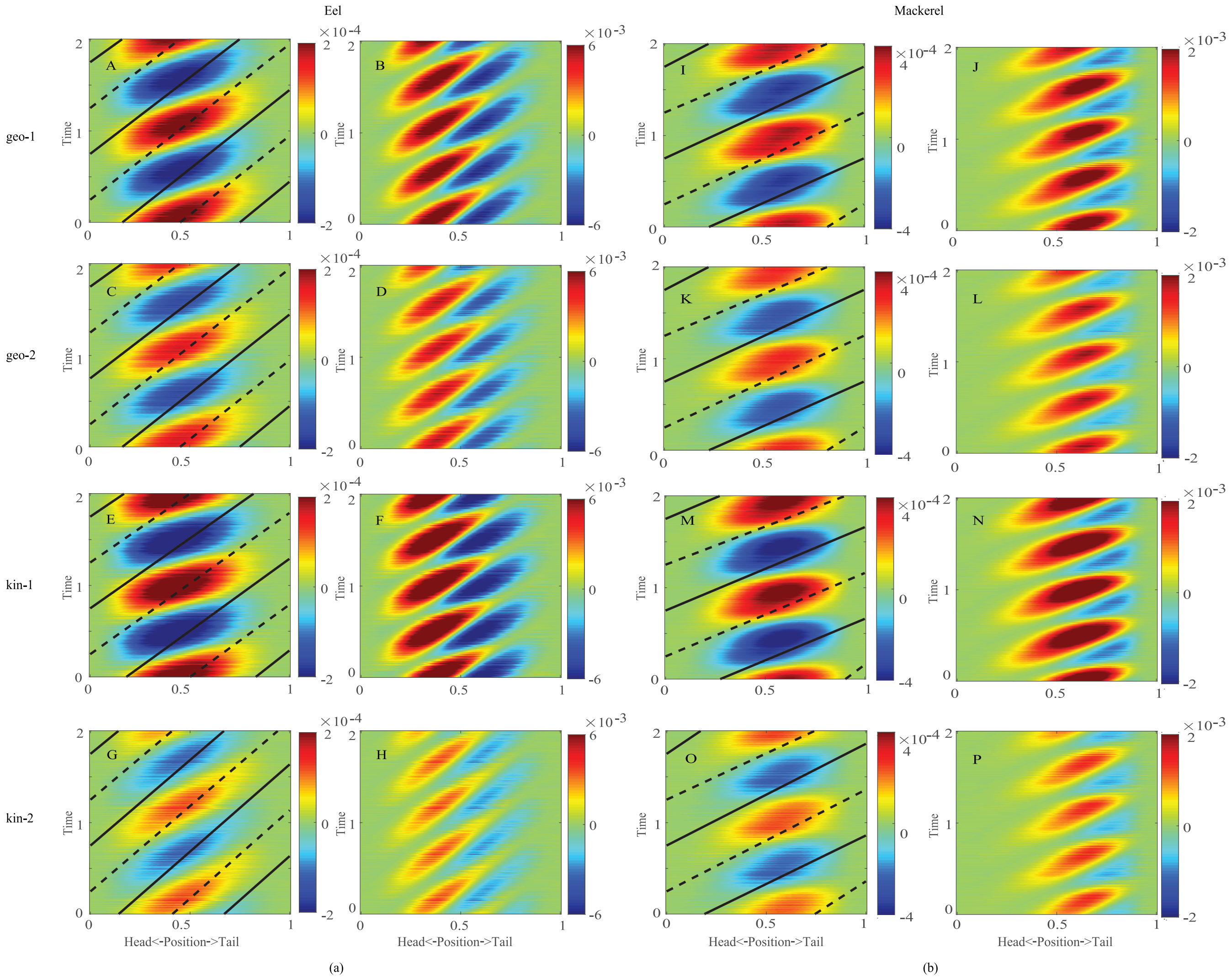

Figure 1: Torque (left column) and power (right column) distributions for eel(a) and mackerel(b) for different cases. All data are collected from the third and forth cycles.

The influence of the mesh refinement

To verify that the mesh size we chose is small enough, we scaled the mesh size of both the fluid and the fish by 0.8. The swimming speed is 0.28, slightly slower than the control case (0.29). Since the value of the torque fluctuates greatly, we filtered the torque before comparison. The differences between the two cases are within the range of error (see S5 Video).

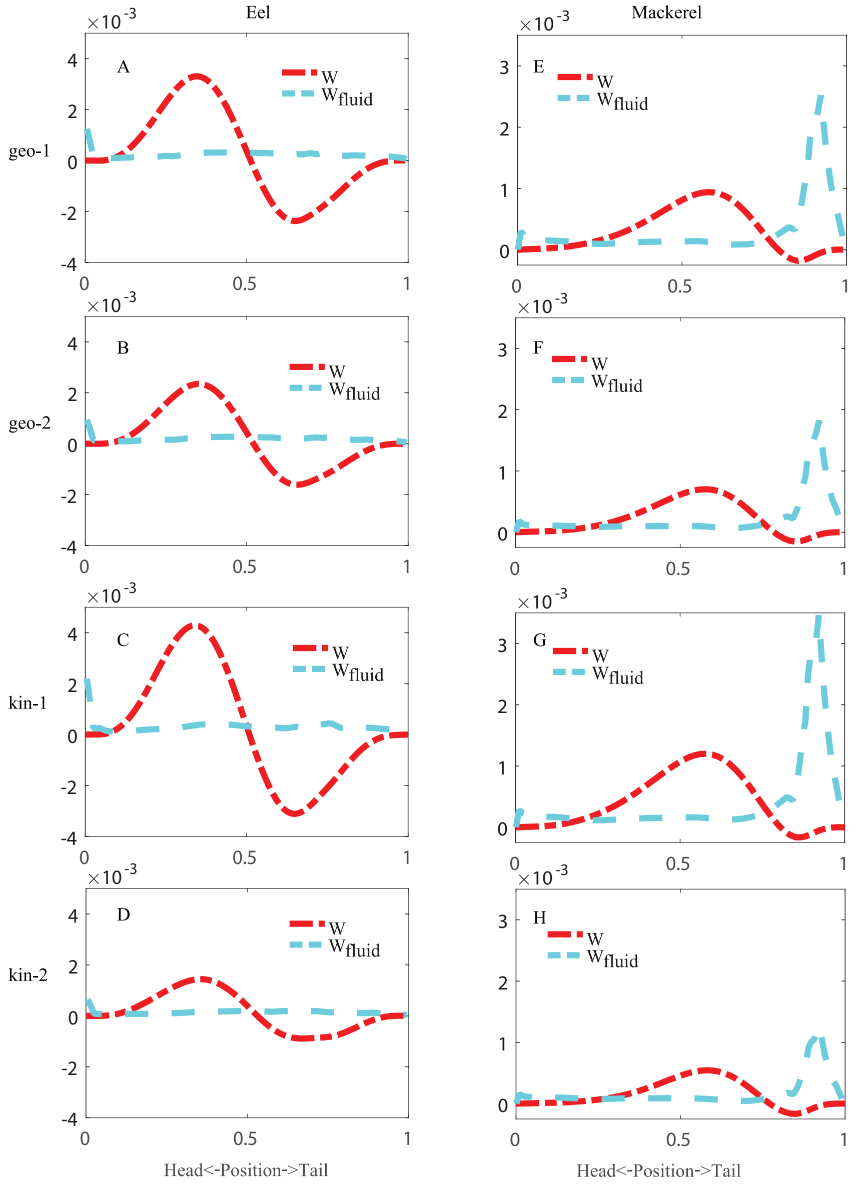

Figure 2: Work distributions for eel (left column) and mackerel (right column) for different cases. All data are collected from the third and forth cycles.

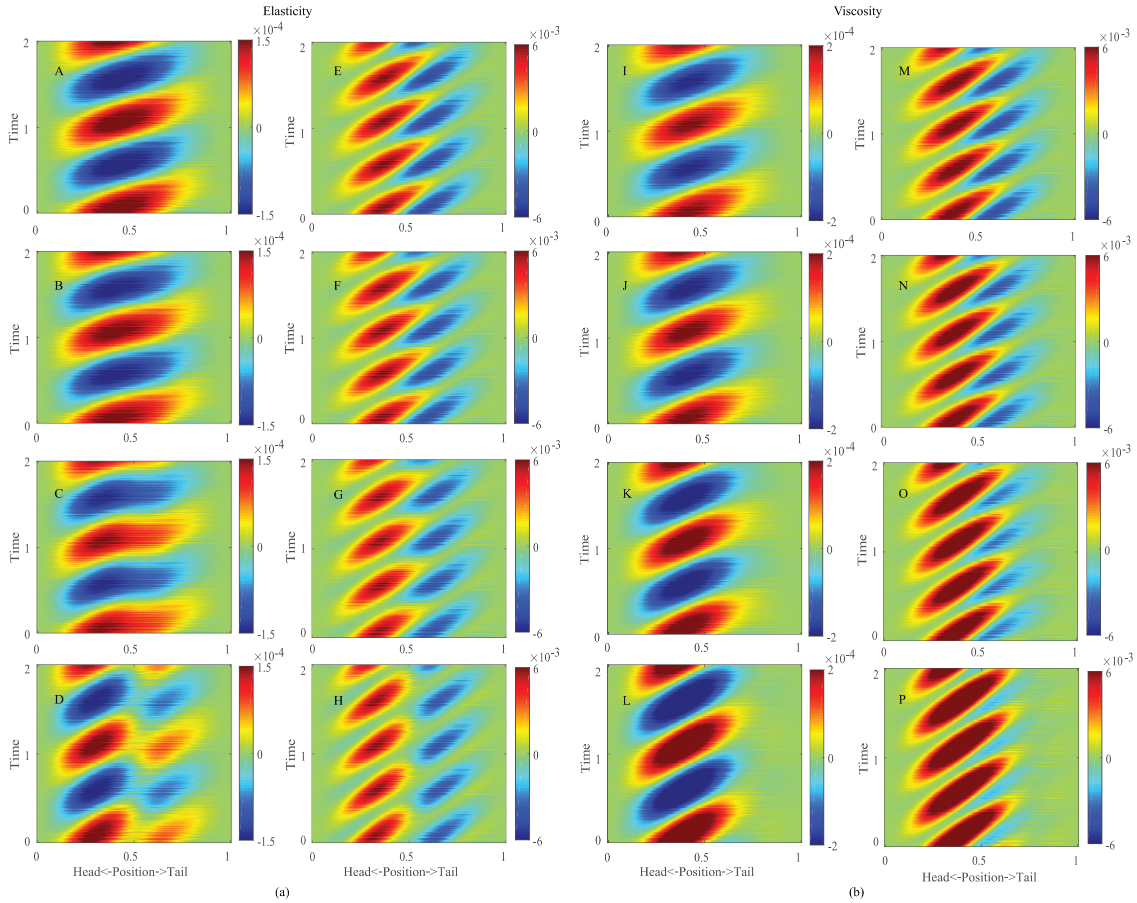

Figure 3: Torque(left column) and power(right column) distributions for eel when elasticity and viscosity are considered. The coefficients of elasticity and viscosity vary from 0.1 to 0.8 from top to bottom. All data are collected from the third and forth cycles.

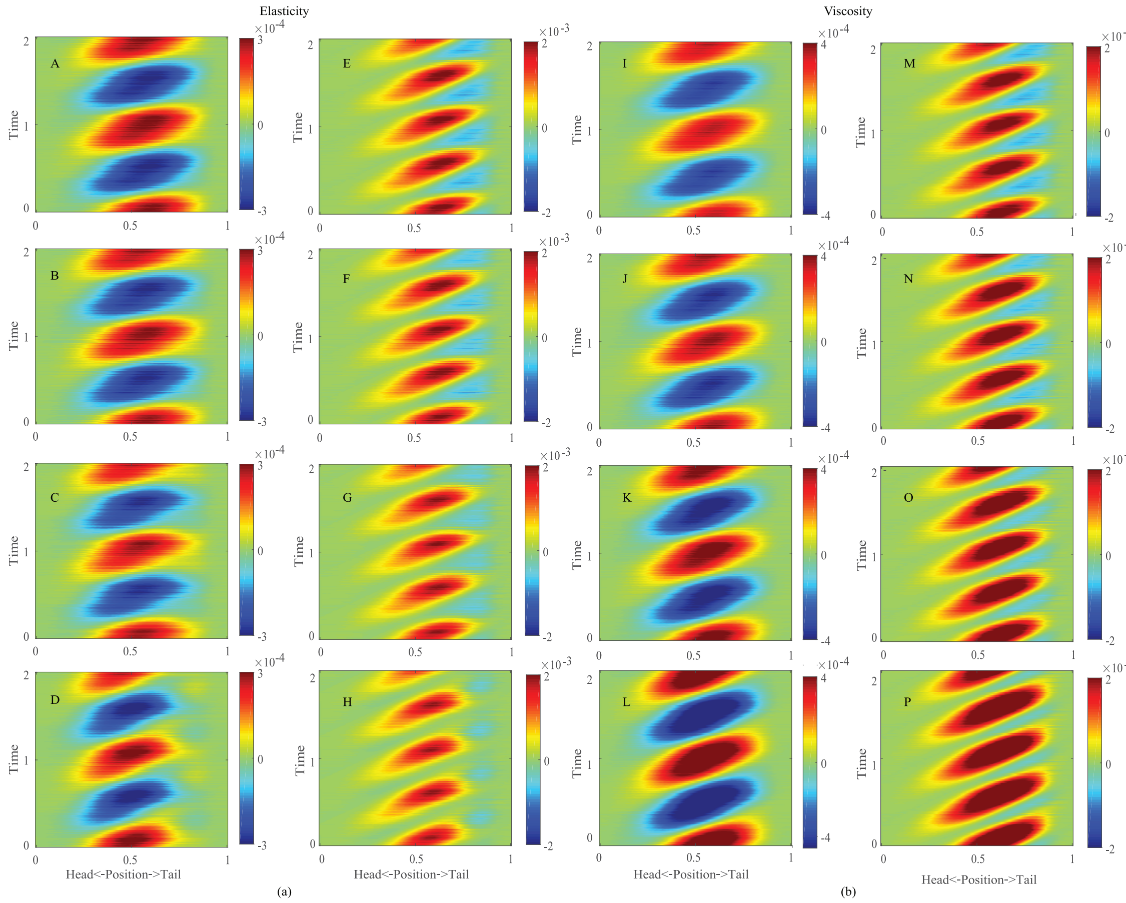

Figure 4: Torque(left column) and power(right column) distributions for mackerel when elasticity and viscosity are considered. The coefficients of elasticity and viscosity vary from 0.1 to 0.8 from top to bottom. All data are collected from the third and forth cycles.

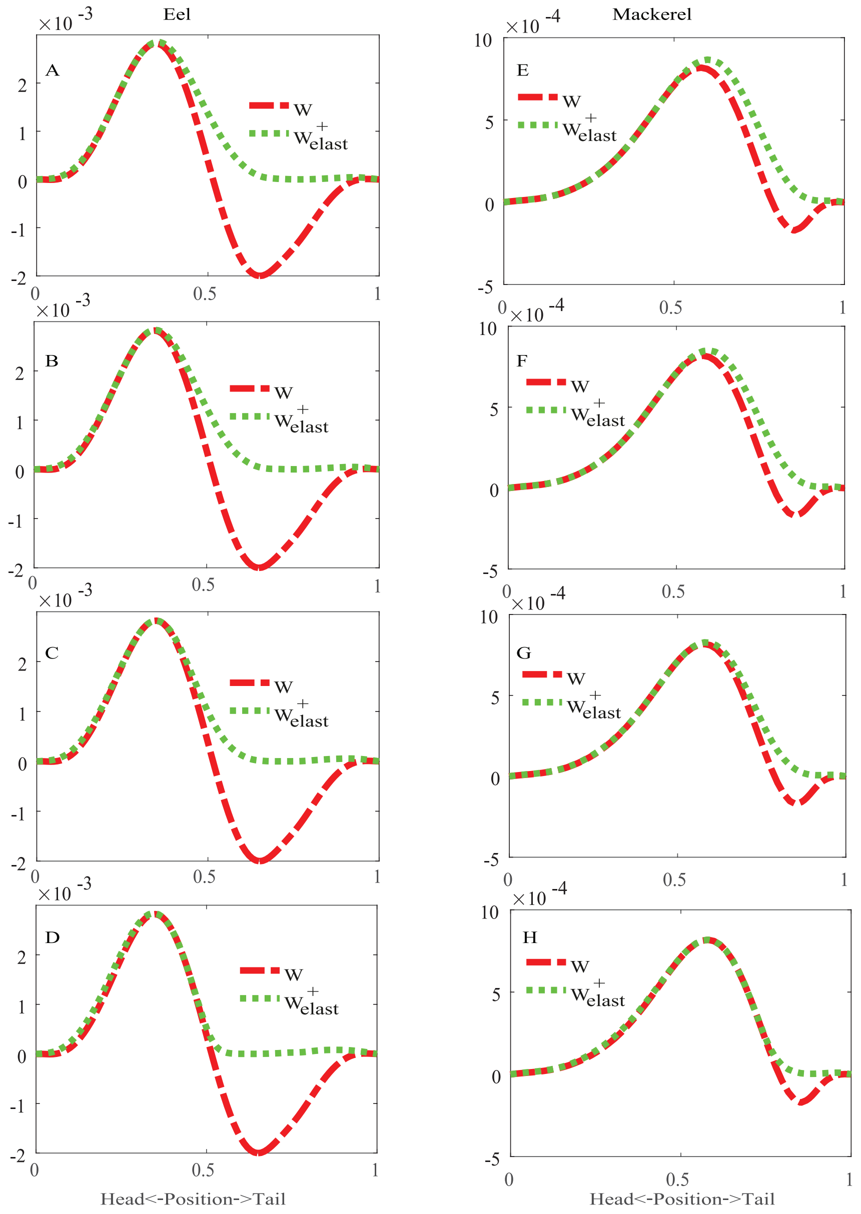

Figure 5: Work distributions for eel(left column) and mackerel(right column) when elasticity is considered. The coefficient of elasticity varys from 0.1 to 0.8 from top to bottom. All data are collected from the third and forth cycles.

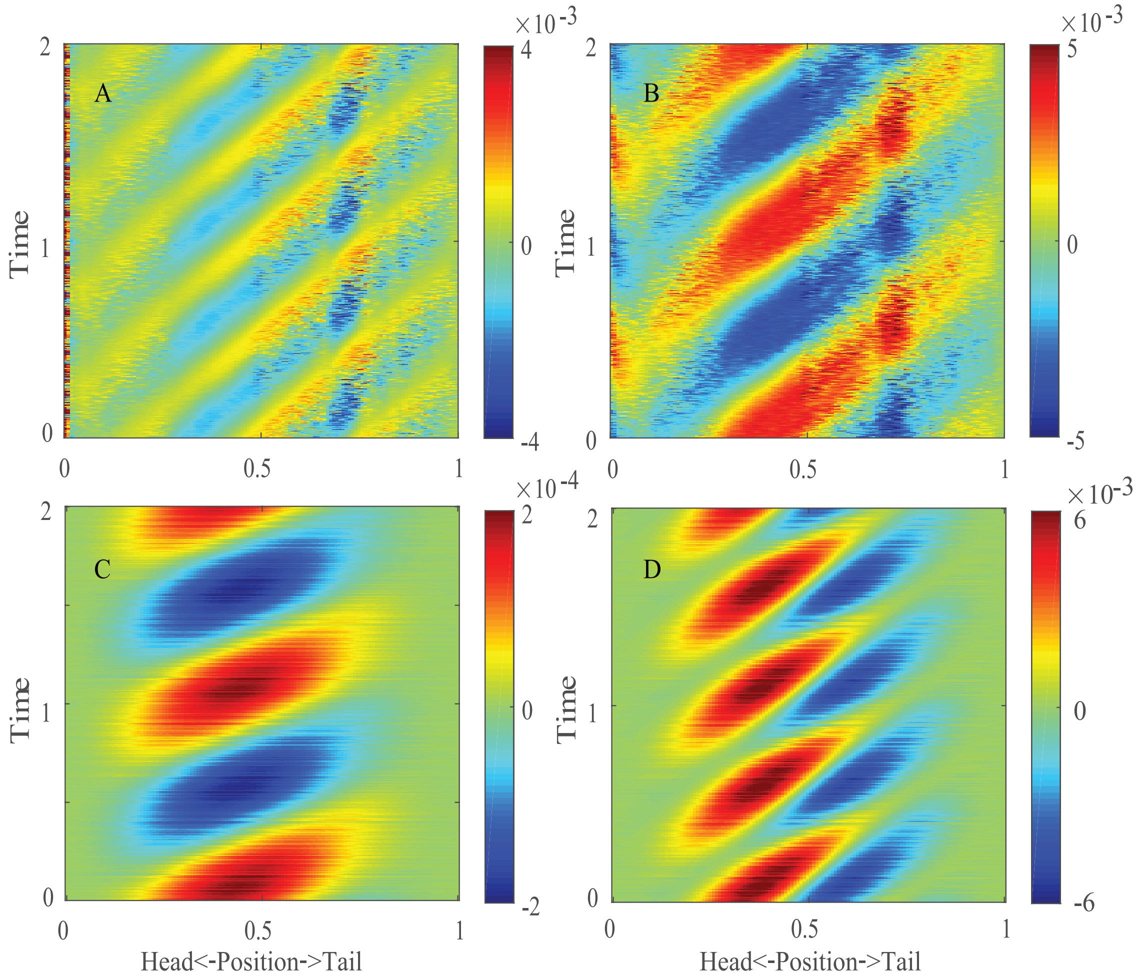

Figure 6: Forward force (A), lateral force (B), torque (C) and power (D) distributions for eel with mesh refinement. All data are collected from the third and forth cycles.
