# Supplementary material for: 3D computational models explain muscle activation patterns and energetic functions of internal structures in fish swimming
Source: PLoS Comput Biol. 2019 Sep 5;15(9):e1006883. doi: 10.1371/journal.pcbi.1006883 (PMC6748450; doi:10.1371/journal.pcbi.1006883)
Supplement: S3 Appendix — (PDF) [file pcbi.1006883.s003.pdf]

Supplementary Information for "3D computational models explain muscle activation patterns and energetic functions of internal structures in fish swimming"

Torque and power patterns from pure resistive forces and reactive forces

To compute the torque and power patterns associated with pure resistive forces and reactive forces, we consider simplified cases which can be solved analytically. We assume that the body is uniform and the curvature wave is prescribed by $\kappa(s,t) = A\sin(2\pi s + 2\pi t)$, where $A \ll 1$, $0 \le s \le 1$, and $0 \le t \le 1$. It is easy to obtain that the lateral motions resulted from resistive forces $\mathbf{F}_d = -C_d\mathbf{v}$ or reactive forces $\mathbf{F}_i = -m\mathbf{a}$ can be both approximated as :

$$y(x,t) = -A\big[\sin(2\pi t + 2\pi x) + \frac{6\left(x - \frac{1}{2}\right)\cos(2\pi t)}{\pi}\big] \tag{S1}$$

The torque associated can be computed as

$$T(s,t) \approx -\int_s^1 xF_d = \int_s^1 xC_d v_y \mathrm{d}x = C_d[\cos(2\pi t) - \cos(2\pi x - 2\pi t) + 2\pi x(1 - 3x + 2x^2)\sin(2\pi t)]/(2\pi), \tag{S2}$$

and

$$T(s,t) \approx -\int_s^1 xF_i = -\int_s^1 xma_y \mathrm{d}x = m[-\sin(2\pi t) - \sin(2\pi x - 2\pi t) + 2\pi x(1 - 3x + 2x^2)\cos(2\pi t)], \tag{S3}$$

,respectively. In the above analysis, m is a constant which represents the added mass per unit length.
