# Supplementary figures and images for: 3D computational models explain muscle activation patterns and energetic functions of internal structures in fish swimming
Source: PLoS Comput Biol. 2019 Sep 5;15(9):e1006883. doi: 10.1371/journal.pcbi.1006883 (PMC6748450; doi:10.1371/journal.pcbi.1006883)

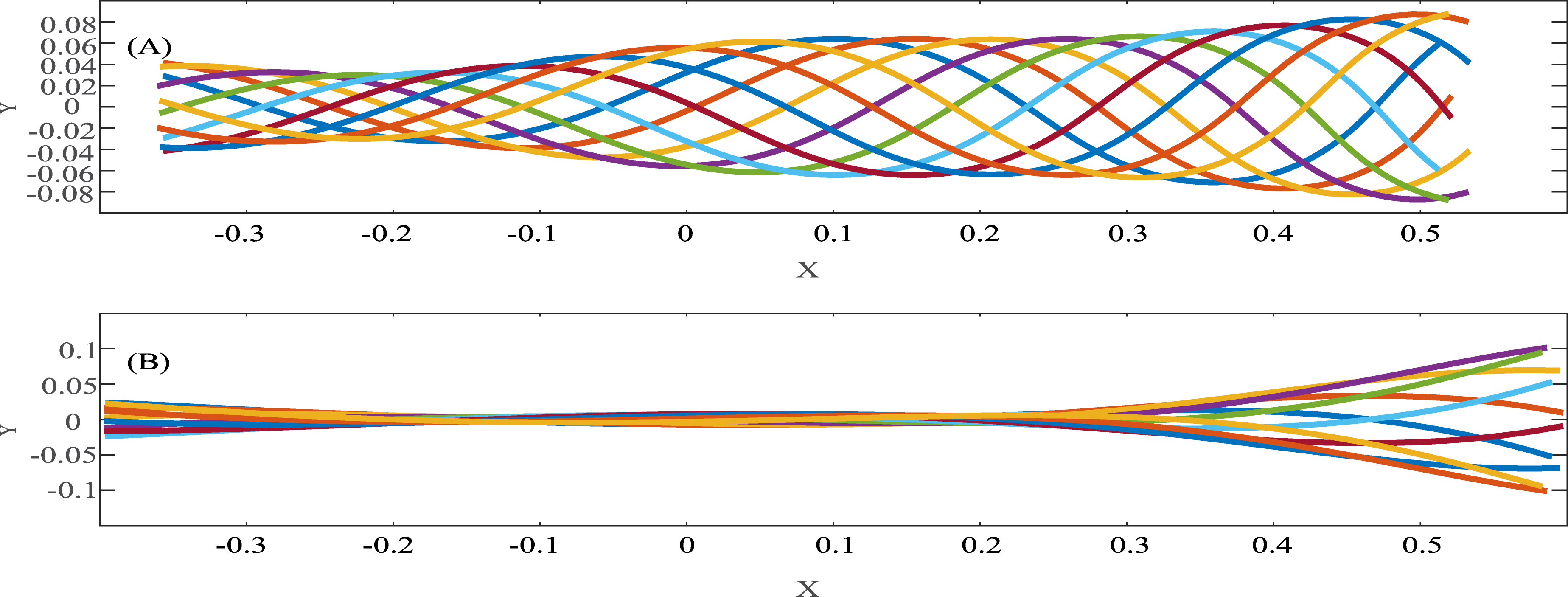

Supplement: S1 Fig — (A) Eel. (B) Mackerel. (TIF) [file pcbi.1006883.s004.tif]
